# Birefringence-derived artifact in optical coherence tomography imaging of the lamina cribrosa in eyes with glaucoma

**DOI:** 10.1038/s41598-023-43820-5

**Published:** 2023-10-11

**Authors:** Masahiro Miura, Shuichi Makita, Yoshiaki Yasuno, Hayate Nakagawa, Shinnosuke Azuma, Toshihiro Mino, Atsuya Miki

**Affiliations:** 1 Department of Ophthalmology, Ibaraki Medical Center, Tokyo Medical University, 3-20-1 Chuo, Ami, Inashiki, Ibaraki 300395 Japan; 2 Computational Optics Group, University of Tsukuba, Tsukuba, Japan; 3 Topcon Corporation, Tokyo, Japan; 4 Department of Myopia Control Research, Aichi Medical University, Nagakude, Japan

**Keywords:** Medical imaging, Glaucoma

## Abstract

We investigated birefringence-derived artifacts that potentially mimic focal defects of the lamina cribrosa (focal LC defects) in optical coherence tomography (OCT) imaging of eyes with glaucoma. This study included 74 eyes of 48 patients with glaucoma. Five horizontal line B-scan images of the optic disc were obtained using commercial swept-source OCT. From a dataset of prototype swept-source polarization-diversity OCT, we calculated the following types of OCT images: polarization-dependent, polarization-dependent attenuation-coefficient, polarization-independent, and polarization-independent attenuation-coefficient. We assessed the commercial OCT images for the presence of birefringence-derived artifacts by comparison with the polarization-diversity OCT images. Commercial OCT showed suggestive findings of focal LC defects in 17 of 74 eyes. Reevaluation using polarization-independent OCT revealed that the focal LC defects in one of 17 eyes (5.9%) were actually birefringence-derived artifacts. This study demonstrated the existence of birefringence-derived artifacts mimicking focal LC defects in commercial OCT imaging and indicated that polarization-diversity OCT is an effective tool to evaluate the presence of these artifacts.

## Introduction

Glaucoma is a progressive optic neuropathy characterized by the degeneration of retinal ganglion cells and their axons, resulting in functional impairment^1^. The lamina cribrosa (LC) has long been considered to be the primary location for axonal damage in glaucoma^1,2^. The LC is a complex, mesh-like structure located within the sclera that serves as the exit point for the axons of retinal ganglion cells from the eye^1^. In eyes with glaucoma, increased intraocular pressure induces LC deformation, including posterior lamina displacement, lamina thinning, pore deformities, and focal defects of the LC (focal LC defects)^3^. Among them, focal LC defects, including laminar holes and laminar disinsertion^4^, are associated with disc hemorrhage^5^, glaucomatous visual field progression^6^, neuroretinal rim thinning^4^, retinal nerve fiber defects^7^, and myopic glaucomatous change^8,9^. Therefore, focal LC defects are considered significant in monitoring glaucoma-associated change in the LC.

Histological research showed that the LC is surrounded by the sclera^10^. The sclera is a robust structure that protects the internal components of the eye against the distension caused by intraocular pressure^11^. It displays birefringence as a result of densely packed collagen layers, and this birefringence causes a modification in the polarization state of the incident light^12,13^. In conventional optical coherence tomography (OCT), the birefringence of the tissue alters the polarization states of the probe beam. Because the OCT signal intensity depends on the mutual relationship of the polarization states of the probe and the reference beams, the tissue birefringence alters the OCT signal intensity. This change in OCT signals can cause artifacts in conventional OCT images^14,15^.

Utilization of the polarization-diversity detection technique, commonly used in polarization-sensitive OCT^16,17^, can eliminate these artifacts originating from birefringence^14,15^. It was reported that commercial OCT images exhibit artifacts with band-like structures caused by the scleral birefringence^18^. Because such artifacts exhibit substantial variability in both their patterns and locations, they may be misinterpreted as scleral vessels. If these birefringence-derived artifacts are formed adjacent to the LC, they may influence its clinical evaluation. Fortunately, these artifactual and real structures can be readily differentiated by utilizing datasets comprising polarization-diversity OCT images^14,15,18^.

The purposes of this study were to assess the artifacts resulting from scleral birefringence adjacent to the LC in eyes with glaucoma using polarization-diversity OCT and commercial OCT and to investigate the clinical relevance of these artifacts in assessing the LC.

## Methods

This prospective, observational, cross-sectional study was approved by the Institutional Review Board of Tokyo Medical University (approval number: T2019-0072). Written informed consent was obtained from each participant before performing any study procedures or examinations. The study was registered in the University Hospital Medical Information Network database (UMIN 000,039,650; http://www.umin.ac.jp/ctr/index-j.htm). This study was performed using a protocol that adhered to the tenets of the Declaration of Helsinki.

We examined 74 eyes of 48 patients (28 men, 20 women; age range, 39–89 years; mean age, 70.2 years) with glaucoma. Diagnosis of glaucoma was based on glaucomatous optic disc appearance (i.e., the presence of focal thinning, notching of the neuroretinal rim, or localized or diffuse atrophy of the retinal nerve fiber layer) and was determined by glaucoma specialists (A.M. and M.M.) by disc photography and abnormal retinal nerve fiber layer profile obtained by commercial OCT (DRI-OCT Triton; Topcon Corp., Tokyo, Japan). If the two specialists did not agree on the glaucoma diagnosis, they reviewed and discussed the data to reach consensus. The glaucoma subtypes were normal tension glaucoma in 21 eyes and primary open angle glaucoma in 53 eyes. Among them, 10 of 21 eyes with normal tension glaucoma and 11 of 53 eyes with primary open angle glaucoma were accompanied by pathologic myopia (axial length of ≥ 26.5 mm).

**Figure 1:**
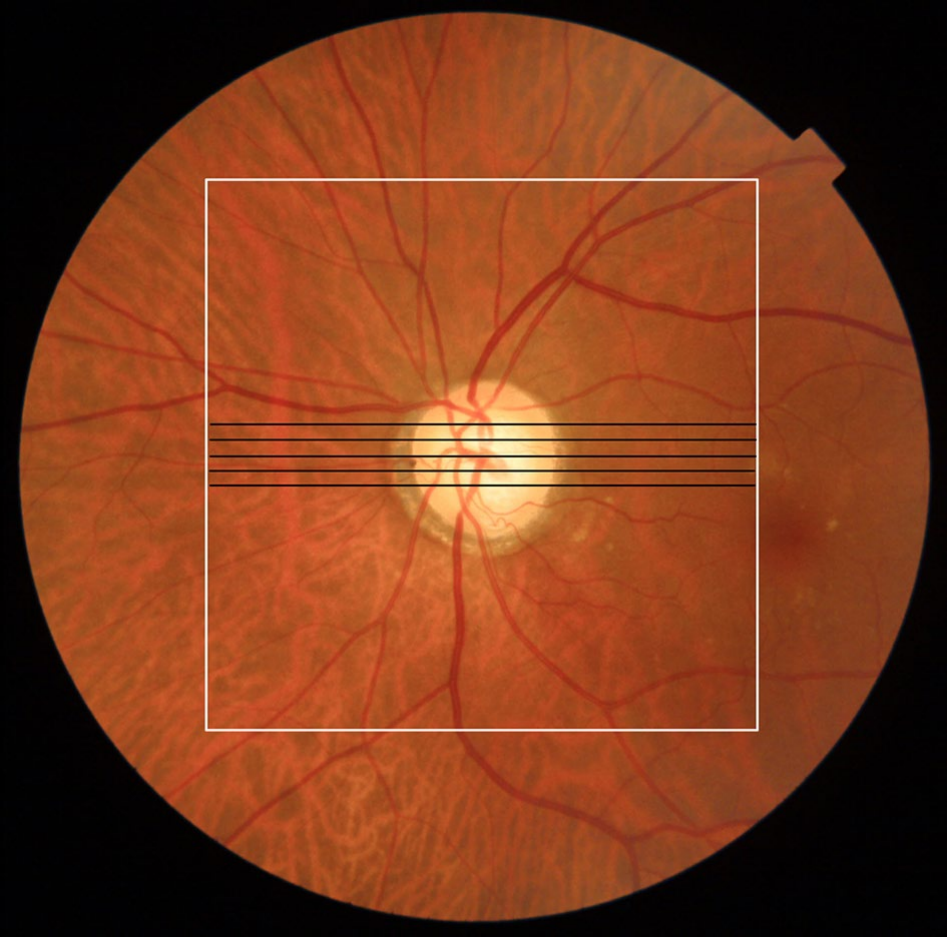
Measurement area in a color fundus image of the left eye of an 84-year-old man with primary open angle glaucoma. The black lines indicate five sets of commercial optical coherence tomography (OCT) B-scan images. The white square indicates the measurement area of polarization-diversity OCT.

Commercial OCT measurement was performed using a DRI-OCT Triton system (Topcon Corp.). This commercial OCT system is a polarization-dependent swept-source OCT device with a 1 μm wavelength band. In this context, polarization-dependent means that the device captures only one of the two polarization components of the probe beam; thus, it is susceptible to birefringence-derived artifacts. The catalog-specified axial resolution was 8 µm in the tissue. The axial scan speed was 100,000 A-scans/s. Five horizontal B-scans of the optic disc were obtained using the five-line cross mode. Each B-scan image consisted of 1,024 A-scans, and the depth range for each B-scan image was 2.6 mm in the tissue. The length of each B-scan was 6 mm, and the vertical interval between each set of B-scan images was 0.15 mm (Fig. 1). Each B-scan image was constructed by averaging 16 consecutive B-scan images.

We calculated two types of scattering OCT images using polarization-diversity OCT: polarization-dependent images and polarization-independent images. A detailed description of the prototype polarization-diversity OCT system was previously published^19^. The polarization-diversity OCT system is a swept-source OCT device with a 1 μm wavelength band and polarization-diversity detection; it enables the measurement of the polarization state of backscattered light from the eye. The axial resolution was measured as 6 µm in the tissue. The axial scan speed was 100,000 A-scans/s, and the depth range of each B-scan was 2.8 mm in the tissue. This polarization-diversity OCT system used an OCT retinal scanner that was modified from the DRI-OCT-1 Atlantis (Topcon Corp.).

Polarization-dependent OCT images were constructed by complex averaging of OCT signals from two detection channels of the polarization diverse detector, corresponding to the two orthogonal polarization components of the detected light [Eq. (3) of Ref. 20]^20^. The complex averaging process mimics the following physical process. The probe beam is split into polarization channels, which are combined in the form of the complex field, and this combined field is identical to the original physical probe beam before splitting. Similarly, this combination process reconstructs the original reference beam. Because the complex averaged signal is the summation of these virtually reconstructed original probe and reference beams, it is identical to the standard OCT signal. Therefore, the complex averaged OCT images exhibit birefringence-derived artifacts similar to the artifacts present in single-detector OCT images (i.e., images from conventional commercial OCT).

Polarization-independent OCT images were constructed by intensity averaging over the two OCT signals Eq. (5) of Ref. 19^19^. When the reference beam powers of the two channels are properly balanced, the intensity of the intensity-averaging signal becomes proportional to the total power of the backscattered probe beam, regardless of the polarization state of the backscattered probe beam. The intensity-averaging signal is thus insensitive to the polarization state variation of the backscattered probe beam. Accordingly, the intensity-averaged OCT images are free of birefringence-derived artifacts.

Evaluating the LC using OCT imaging is hindered by shadow artifacts caused by signal attenuation arising from the central retinal vessel trunk, significantly reducing the visibility of deep structures^21^. To mitigate the LC structure obscurity caused by shadow artifacts, we calculated attenuation-coefficient (AC) images using Eq. (17) of Ref. 22^22^, which represents the rate of attenuation of the OCT signal intensity at each depth. Note that this does not correspond to the optical attenuation coefficient of tissues because it does not account for systematic effects on signal attenuation^23^. We adjusted the display range of the attenuation coefficient in grayscale B-scan images from 0.14 to 54.60 mm^−1^ to optimize the visibility of the LC structure. Consequently, we calculated polarization-dependent AC-OCT images and polarization-independent AC-OCT images from the polarization-dependent and polarization-independent OCT images, respectively.

We conducted volumetric scans using a raster scanning protocol with 512 A-lines × 256 B-scans covering a 6.0 × 6.0 mm region of the retina (Fig. 1). From a series of 256 B-scan images of polarization-diversity OCT images, we manually selected polarization-diversity OCT B-scan images at locations corresponding to five sets of commercial OCT images.

Subsequently, birefringence-derived artifacts in OCT images of the optic disc were evaluated using commercial OCT images and polarization-diversity OCT images. In the first evaluation, two ophthalmologists (A.M. and H.N.) subjectively evaluated the presence of focal LC defects in the commercial OCT images while also referring to the polarization-dependent AC-OCT images and the polarization-dependent OCT images. In this evaluation, the polarization-dependent OCT images were used to confirm the consistency of the location between commercial OCT images and polarization-diversity OCT images. The second evaluation investigated the presence of birefringence-derived artifacts mimicking focal LC defects in commercial OCT imaging. In this evaluation, commercial OCT B-scan images with focal LC defects were reevaluated by two ophthalmologists (A.M. and H.N.) with reference to polarization-independent AC-OCT images and polarization-independent OCT images. Cases of disagreement concerning the first and second evaluations were resolved by consensus after discussion with a third ophthalmologist (M.M.).

To evaluate the focal LC defects in *enface* images of the LC, *enface* images were reconstructed from the volumetric data of polarization-diversity OCT. We compared the *enface* images of polarization-dependent and polarization-independent AC-OCT, using polarization-diversity OCT volumes without significant motion artifacts for this comparison.

Statistical analyses were performed using IBM SPSS Statistics for Windows, version 28.0 (IBM Corp., Armonk, NY, USA).

## Results

**Figure 2:**
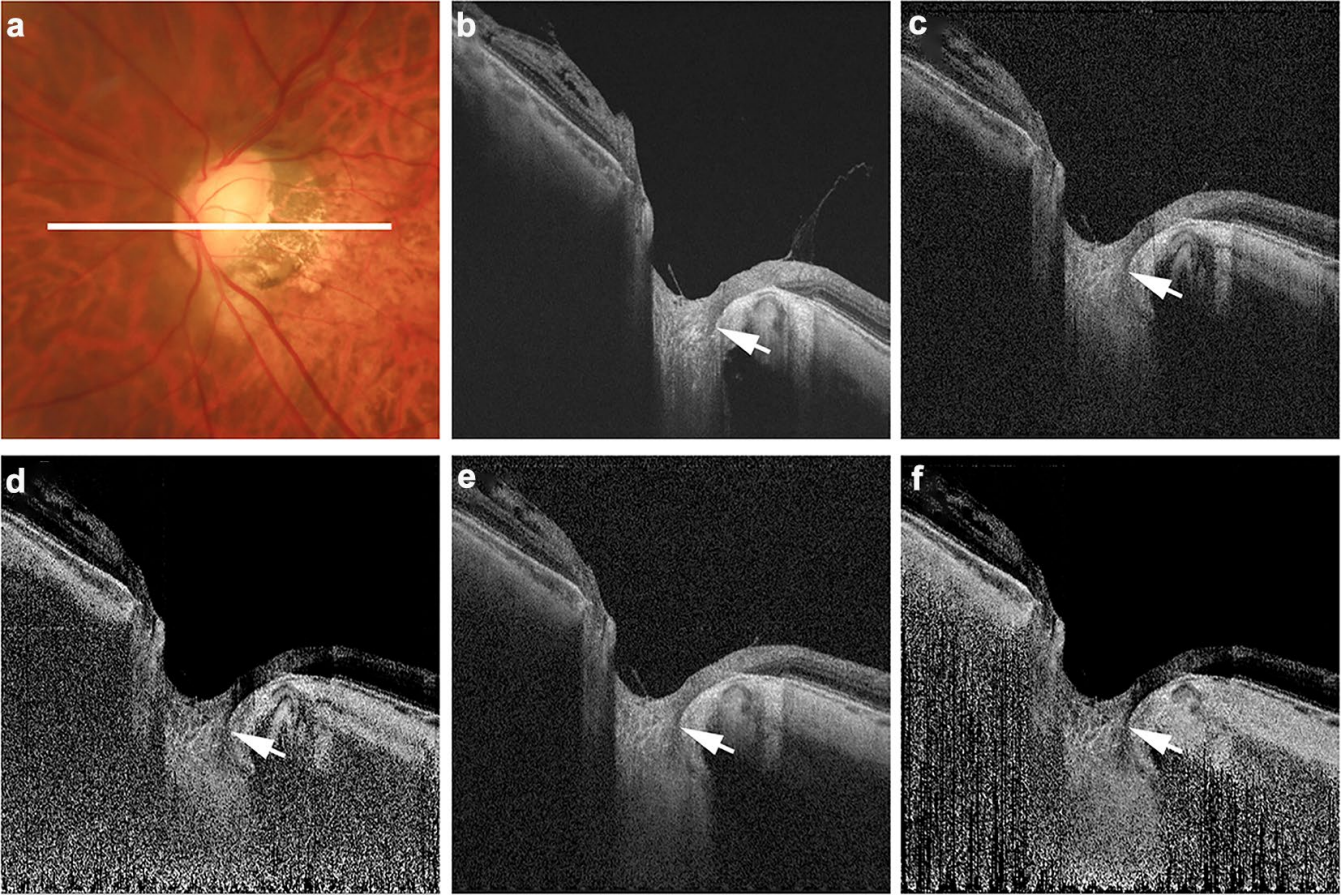
Real laminar disinsertion without artifactual structures in commercial optical coherence tomography (OCT) image and polarization-diversity OCT images. The left eye of a 56-year-old woman with normal tension glaucoma in conjunction with pathologic myopia (axial length: 29.0 mm) is shown. The white line in the color fundus image (**a**) indicates the scan line for the commercial OCT image and polarization-diversity OCT B-scan images (**b**–**f**). The commercial OCT B-scan image (**b**) showed laminar disinsertion (white arrow). Each polarization-diversity OCT B-scan image [polarization-dependent OCT image (**c**), polarization-dependent attenuation-coefficient OCT image (**d**), polarization-independent OCT image (**e**), polarization-independent attenuation-coefficient OCT image (**f**)] showed laminar disinsertion at the same location (white arrow).

**Figure 3:**
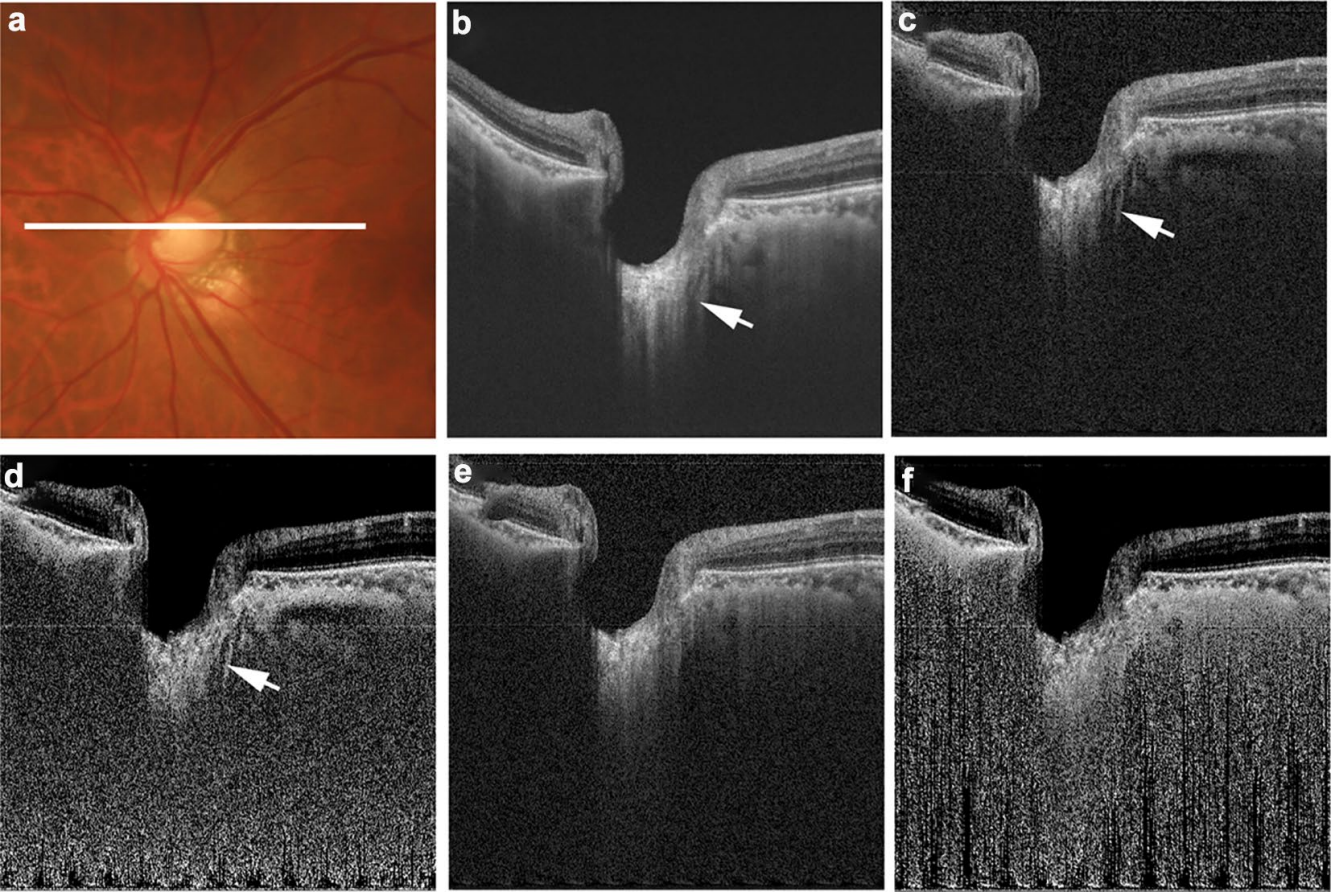
Birefringence-derived artifact resembling laminar disinsertion in commercial optical coherence tomography (OCT) image and polarization-diversity OCT images. The left eye of a 62-year-old man with primary open angle glaucoma in conjunction with pathologic myopia (axial length: 27.3 mm) is shown. The white line in the color fundus image (**a**) indicates the scan line for the commercial OCT image and polarization-diversity OCT B-scan images (**b**–**f**). The commercial OCT B-scan image (**b**) shows a structure resembling laminar disinsertion (white arrow). Both the polarization-dependent OCT image (**c**) and the polarization-dependent attenuation-coefficient OCT image (**d**) also show a structure resembling laminar disinsertion at the same location (white arrows). By contrast, neither the polarization-independent OCT image (**e**) nor the polarization-independent attenuation-coefficient OCT image (**d**) show structures resembling laminar disinsertion.

In the first evaluation, focal LC defects were detected in 17 of 74 eyes (23.0%) or 30 of 370 B-scan images (8.1%) using commercial OCT B-scan images. The subtypes of focal LC defects were laminar hole in one eye (four B-scan images) and laminar disinsertion in 16 eyes (26 B-scan images). The second evaluation of these 17 eyes confirmed the presence of a birefringence-derived artifact mimicking laminar disinsertion in one eye (two B-scan images) in the commercial OCT B-scan images. Consequently, the focal LC defects in commercial OCT images were classified as artifacts in one of 17 eyes (5.9%) or two of 30 B-scan images (6.7%). Figure 2 presents a representative case of real laminar disinsertion. Real laminar disinsertion was confirmed in all types of B-scan images in commercial OCT and polarization-diversity OCT (polarization-dependent OCT, polarization-dependent AC-OCT, polarization-independent OCT, and polarization-independent AC-OCT). Figure 3 shows a birefringence-derived artifact mimicking laminar disinsertion. This artifactual dark band is arranged in a nearly vertical direction at a location adjacent to the LC and was observed in commercial OCT, polarization-dependent OCT, and polarization-dependent AC-OCT images. By contrast, polarization-independent OCT and polarization-independent AC-OCT images did not display this artifact. Using polarization-diversity OCT imaging, we confirmed that this finding that mimics laminar disinsertion is actually a birefringence-derived artifact.

Regarding the first evaluation by the specialists, the concordance rate (kappa value) was 0.80 (*p* < 0.001). In the second evaluation, the two specialists agreed on all B-scan images.

**Figure 4:**
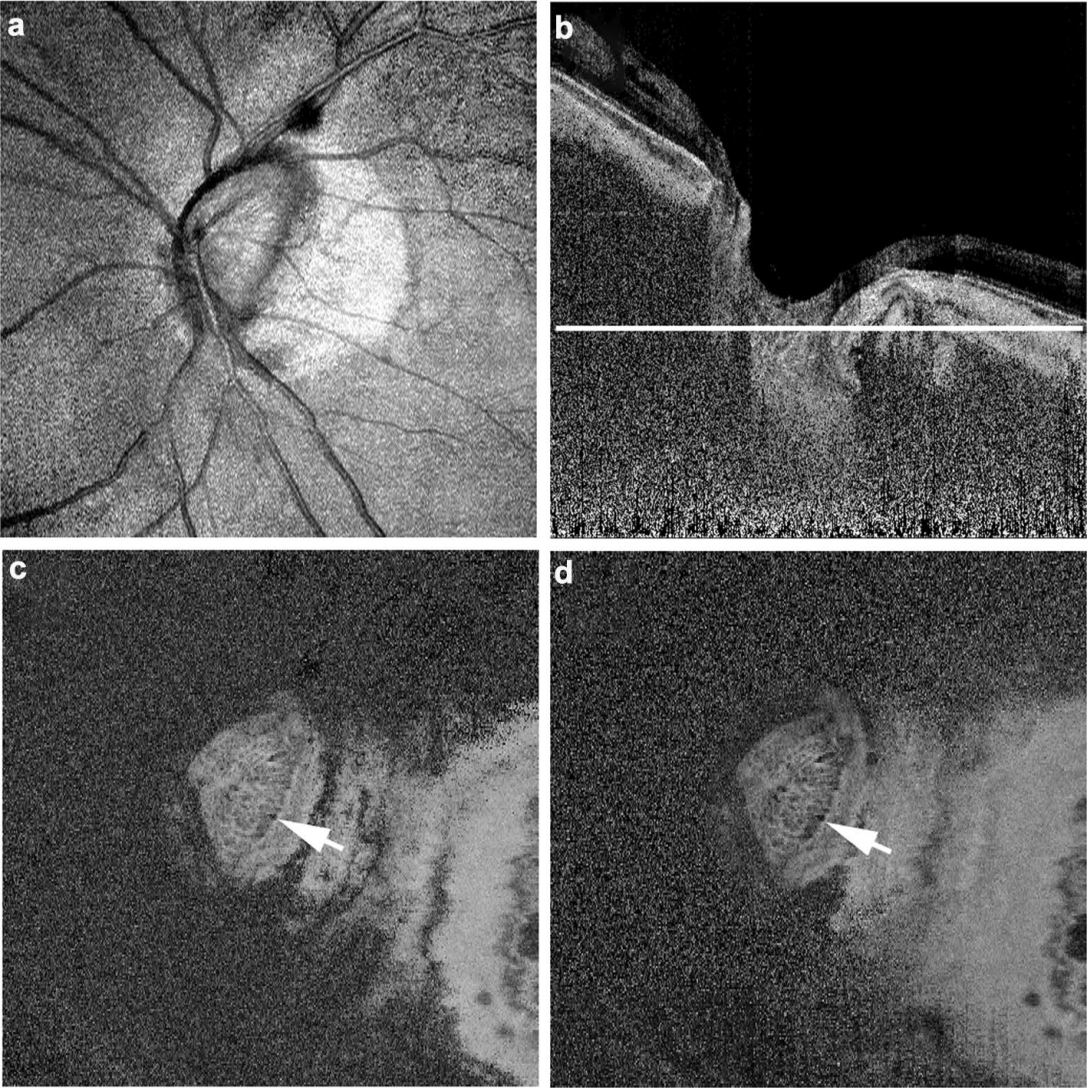
*Enface* polarization-diversity optical coherence tomography (OCT) images at the level of the lamina cribrosa of the eye in Fig. 2. Projection image of polarization-dependent OCT (**a**). The white line in the polarization-dependent attenuation-coefficient B-scan OCT image (**b**) shows the scan line of *enface* OCT images (**c**,**d**). *Enface* images reconstructed from the volume data of the polarization-dependent attenuation-coefficient OCT image (**c**) and the polarization-independent attenuation-coefficient OCT image (**d**) show the real laminar disinsertion as a low-intensity area (white arrow).

**Figure 5:**
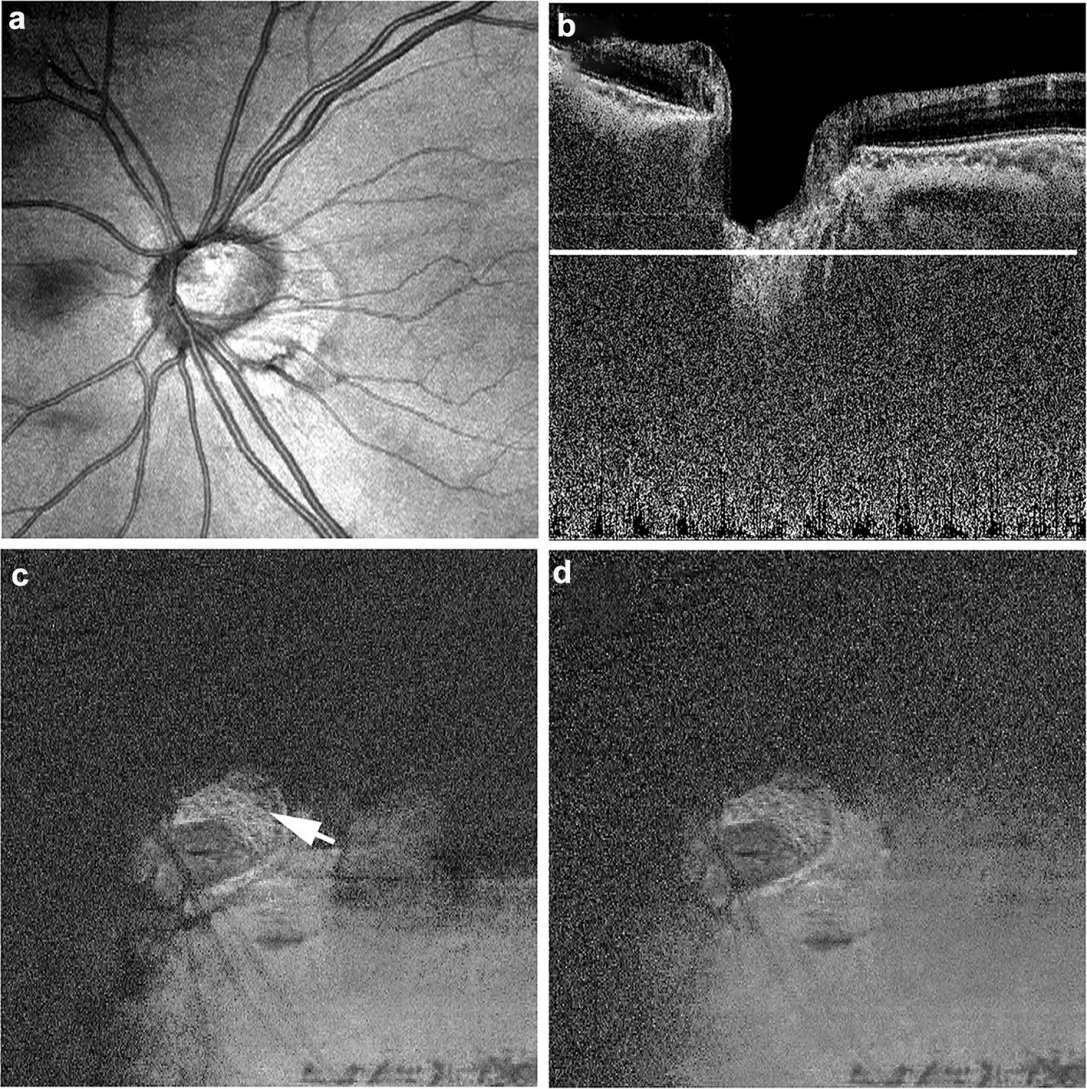
*Enface* polarization-diversity optical coherence tomography (OCT) images at the level of the lamina cribrosa of the eye in Fig. 3. Projection image of polarization-dependent OCT (**a**). The white line in the polarization-dependent attenuation-coefficient B-scan OCT image (**b**) shows the scan line of *enface* OCT images (**c**,**d**). The *enface* image reconstructed from the volume data of the polarization-dependent attenuation-coefficient OCT image (**c**) shows an artifactual structure resembling laminar disinsertion as a low-intensity area (white arrow). By contrast, the *enface* image of the polarization-independent attenuation-coefficient OCT image (**d**) does not show this low-intensity area.

In *enface* images of the LC, real laminar disinsertion was observed as a low-intensity area adjacent to the LC in both polarization-dependent AC-OCT images and polarization-independent AC-OCT images (Fig. 4). By contrast, birefringence-derived artifacts were observed as a low-intensity area only in the polarization-dependent AC-OCT image. The polarization-independent AC-OCT *enface* image did not display this low-intensity area (Fig. 5).

## Discussion

Analysis of glaucoma using OCT images of the LC is becoming increasingly popular^3^. In the present study, we identified a birefringence-derived artifact mimicking laminar disinsertion in commercial OCT imaging. Commercial OCT led to 30 B-scan findings suggestive of laminar disinsertion in 17 of 74 eyes. Among them, reevaluation with polarization-independent OCT revealed that two B-scan findings in 1 of 17 eyes (5.9%) corresponded to birefringence-derived artifacts.

This study demonstrated the existence of birefringence-derived artifacts that mimic laminar disinsertions. Previous research of scleral OCT imaging of pathologic myopia reported the presence of a birefringence-derived artifactual band-like structure resembling scleral vessels^18^. Commercial OCT showed this artifact with considerable diversity in its direction, pattern, and location^18^. The presence of structural birefringence in the sclera is associated with factors such as collagen fibril thickness, diameter, and orientation^13,24,25^. The collagen fiber architecture of the posterior human sclera demonstrates significant anisotropy and inhomogeneity^10^. Fiber anisotropy in the peripapillary sclera is 37% higher than that in the mid-posterior sclera^10^. The intricate nature of scleral collagen fibrils surrounding the optic disc results in a diverse composition of birefringence-derived artifacts characterized by their heterogeneity. If these artifacts are located adjacent to the LC, they may mimic laminar disinsertion.

In a study of pathologic myopia, scleral vessel artifacts were observed in 17 of 76 eyes (22.4%) among the five sets of commercial OCT B-scan images^18^. The incident rate of laminar disinsertion artifacts (1 of 74 eyes; 1.4%) was significantly lower than that of scleral vessel artifacts (*p* < 0.001, Fisher’s exact test). A laminar disinsertion artifact indicates an artifactual band-like structure (i.e., scleral vessel artifact) located around the border of the LC in a nearly vertical direction. Therefore, the laminar disinsertion artifact is considered a particular variation of the scleral vessel artifact. Consequently, the incident rate of laminar disinsertion artifacts was considerably lower than that of scleral vessel artifacts.

Despite the low incident rate of focal LC defect artifacts, it is necessary to consider the potential for artifacts in OCT imaging during clinical examinations. Previous studies have suggested the importance of focal LC defects in glaucoma^3^. The transient changes in translaminar pressure trigger constant remodeling of the LC in response to physiologic and pathophysiologic stimuli^3^. This can potentially lead to pathological remodeling of the structure, including focal LC defects^1^. Focal LC defects are recognized as crucial structural characteristics in glaucoma and have the potential to serve as biomarkers for LC deformity in glaucoma^4–8^. The present study revealed the existence of artifacts arising from birefringence and mimicking focal LC defects in commercial OCT images. To the best of our knowledge, currently available commercial OCT systems for retinal imaging do not employ polarization-diversity detection. Instead, they detect a polarization component that matches the polarization state of the reference beam. Therefore, commercial OCT imaging does not discriminate this artifact from real focal LC defects. These artifacts have the potential to lead to overestimation of focal LC defects and misinterpretation of the underlying pathogenesis of concurrent lesions in glaucoma patients.

The present study had certain limitations. First, given the low number of patients in our sample, we could only examine specific aspects of artifacts arising from birefringence in OCT imaging of the optic disc. Second, we conducted the commercial OCT and polarization-diversity OCT measurements independently, potentially causing inconsistent locations between polarization-diversity OCT B-scan images and commercial OCT B-scan images. This inconsistency may have influenced the detection of artifacts in this study. Third, the polarization-diversity OCT system utilized in this study was unable to quantify birefringence around the optic disc. Measurement of local birefringence is reportedly necessary to comprehensively evaluate the influence of scleral birefringence^24,25^. Fourth, we employed distinct scanning protocols (density and number of B-scans for averaging) for both the commercial OCT and polarization-diversity OCT systems. The disparities resulting from these differences may have impacted the visibility of the LC and consequently could have influenced the comparison between the images obtained from the commercial OCT and polarization-diversity OCT systems. Fifth, previous OCT studies on LC defects were performed using swept-source OCT^8,9^ or spectral-domain OCT^4–7^. In the present study, the DRI-OCT Triton device was used as a representative of commercial swept-source OCT systems; however, other swept-source OCT systems are also commercially available. Because of variations in polarization state optimization, interferometer architecture, and image processing methods, the artifacts resulting from birefringence may exhibit differences among alternative systems. Each commercial OCT system needs to be individually assessed to determine how it is affected by artifacts arising from birefringence. Sixth, several additional factors, such as the angle of incidence and the polarization state of the incident light, may affect the occurrence of birefringence-derived artifacts. In detail, scleral birefringence can be influenced by the relative orientation of the beam and the sample^26,27^. In addition, the occurrence of the artifacts can vary among individual devices because the polarization states of the probe beam and references are not fully controlled^28^. In forthcoming investigation, evaluation of inter-device variation and repeatability of the birefringence-derived artifact could provide more comprehensive information about its clinical significance. Seventh, manual selection was employed to choose polarization-diversity OCT images from the volumetric scan that corresponded to specific locations depicted in the commercial OCT images. The resource restriction imposed by this manual procedure allowed us to evaluate only sets of five B-scan images for each eye. In future studies, performing a volumetric assessment of commercial OCT images in relation to birefringence-derived artifacts could yield more comprehensive data concerning artificial structures within the optic disc.

To summarize, this study established the presence of birefringence-derived artifacts resembling focal LC defects in commercial OCT images. In patients with glaucoma, these artifacts can lead to an overestimation of focal LC defects and result in incorrect interpretation of the configuration change in the LC. In polarization-diversity OCT images, artifacts can be readily differentiated from real structures. Therefore, incorporating a polarization-diversity OCT system has the potential to enhance the precision of OCT imaging when examining the optic disc.

## Data Availability

The datasets used and/or analyzed in the current study are available from the corresponding author upon reasonable request.
